# Risk factors for discontinuing oral immunotherapy in children with persistent cow milk allergy

**DOI:** 10.1002/iid3.668

**Published:** 2022-06-20

**Authors:** Elisa Benelli, Andrea Trombetta, Laura Badina, Stefanny Andrade, Giulia Zamagni, Antonio Prisco, Eugenio Traini, Egidio Barbi, Irene Berti

**Affiliations:** ^1^ Department of Pediatrics Ca'Foncello Hospital Treviso Italy; ^2^ University of Trieste Trieste Italy; ^3^ Institute for Maternal and Child Health IRCCS “Burlo Garofolo” Trieste Italy; ^4^ Department of Pediatrics Università degli Studi della Campania Luigi Vanvitelli Napoli Italy

**Keywords:** cow milk allergy, oral immunotherapy, risk factors

## Abstract

**Background:**

There are no universally accepted criteria for discontinuing milk oral immunotherapy (MOIT) in patients with persistent cow milk allergy (CMA) and little data are available on predictive risk factors for dropping out from oral immunotherapy (OIT), due to allergic reactions or other reasons.

**Methods:**

We retrospectively reviewed clinical records of patients with persistent severe CMA undergoing MOIT in a tertiary care center hospital to investigate risk factors associated with discontinuation of OIT. Persistent and severe allergy was defined as the history of systemic reactions and any milk protein‐specific IgE level >85 kU/ml. All patients were first admitted for an in‐hospital rush phase eventually followed by an at‐home dose increase. We evaluated the effect of various factors on two primary outcomes: the highest dose of milk ingested during the in‐hospital rush phase and during the home OIT phase.

**Results:**

We identified 391 patients, of whom 131 met the inclusion criteria for the retrospective study, 54 females and 77 males. Data of the home OIT phase were available for 104 patients (27%).

Regarding the home OIT outcome, an association for having a cow milk avoiding diet was found with reaching a dose below 10 ml during the in‐hospital rush phase (relative risks [RR]: 2.33, confidence interval [CI]: 0.85; 6.42), an age above than 10 years from the time of admission (RR: 3.29, CI: 0.85; 12.73), and a higher total number of reactions occurred during the hospitalization (RR: 1.54, CI: 1.02; 2.32), whereas the presence of respiratory reactions with wheezing (RR: 1.93, CI: 0.49; 7.61) and an IM adrenaline use was related to a higher risk of having an OIT still in progress (RR: 5.47, CI: 0.33; 7.73).

**Conclusions:**

In this cohort of children with persistent CMA undergoing OIT who presented with respiratory reactions with wheezing, the development of anaphylaxis with the need for IM adrenaline, and age above 10 years were predictors of poor long‐term outcome.

## Key Messages

- This study shows that wheezing reactions during the in‐hospital phase admission, discharge with a dose below 10 ml, use of IM epinephrine, and older age (over 10 years) were all risk factors for OIT discontinuation.
- The provided phenotype of patients with a severe milk allergy who are more likely to fail OIT can help pediatricians to provide to their parents the elements to properly decide the most appropriate approach.

## Graphical abstract

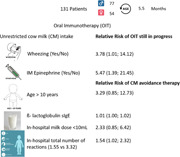

## 1 INTRODUCTION

Food allergy affects approximately 4% of the pediatric population in developed countries.^1^ Cow milk allergy (CMA) is the most common food allergy, with a generally good prognosis, due to the high spontaneous resolution rate as the child grows.^2^ However, a significant percentage of patients do not outgrow their allergy and remain at risk of anaphylaxis, with a relevant impact on their life quality and the potential risk of life‐threatening reactions.

Oral immunotherapy (OIT), which consists of the oral administration of increasing doses of the allergen to gradually desensitize subjects with food allergy, is the only active therapy currently available that could modify the anaphylactic risk of these patients.^3^, ^4^ A recently published guideline suggests OIT for treating patients who do not spontaneously acquire tolerance at 4–5 years.^4^ Although effective in a significant percentage of cases, OIT has limitations both in terms of effectiveness and safety,^5^ and 20% of children with CMA are reported to discontinue treatment due to the significant side effects.^6^ Furthermore, the process is often long and can impact the patients' and families' life quality, with variable outcomes over time.^7^ Few studies specifically investigated on criteria for the discontinuation of milk OIT.^8^, ^9^ An early prediction and detection of patients with a high risk of presenting severe reactions, eventually suspending OIT would be welcome.

This study investigates the risk factors associated with the inability to follow an OIT protocol in patients with severe persistent CMA.

## 2 MATERIAL AND METHODS

We performed a retrospective review of patients with severe CMA who underwent OIT at the Pediatric Clinic of Trieste from January 2008 to January 2017. The study was approved by the Institutional Review Board, IRB 05/17. All parents of the children enrolled signed informed consent for the OIT protocol and anonymous data use.

We defined severe persistent CMA patients as those who, at the time of admission, met all the following criteria:

− age ≥5 years;
− IgE‐specific (sIgE) levels (CAP System; Pharmacia & Upjohn AB Diagnostics) for at least one major CM allergen (α‐lactalbumin, β‐lactoglobulin, casein) ≥85 kU/A;
− According to the Clark and Ewan^10^ classification, a compelling history for at least one severe allergic reaction (i.e., reactions defined as Classes 4 and 5 according to Clark's classification) after accidental exposure to milk or dairy products requiring emergency treatment or a positive open oral food challenge.

Data regarding the onset of allergy (age and symptoms at the onset), history before starting OIT (number and severity of accidental reactions presented by the patient during avoidance diet, use of self‐injectable epinephrine, allergic comorbidity), allergic tests at the time of admission (skin prick test [SPT], total and sIgE and IgG_4_ values), previous treatments were collected. We gathered data about adverse effects and the maximum milk dosage ingested during the “rush” phase in the hospital and during the long‐term home OIT (build‐up and maintenance phase).

### 2.1 Study protocol

This specific oral tolerance induction protocol consists of two phases. After a first in‐hospital rush phase of 10 days, empirically targeted to a maximum amount of 20 ml of pure milk, children are discharged with a slow increasing phase starting from the maximum tolerated CM dosage^10^ (Tables 1 and Supporting Information: Table 2S). Both the in‐hospital and home increase phase are flexible, adapting the milk increase to the patients’ reactions and his/her attitude toward milk intake. The home phase may take from months to years. Antihistamine treatment is usually prescribed in the first months of OIT and eventually weaned. All decisions on milk dose are taken by the allergologist and shared with the family and the child. A 24‐h dedicated phone availability is offered to all families.

To identify the effect of the various risk factors, we divided patients into two categories based on their CMA reactivity thresholds:

(1) A final dose of milk at the time of hospital discharge, arbitrarily establishing two subcategories (patients allowed to reach a final dose of milk lower than 10 ml, patients allowed to reach a final dose of milk ≥10 ml).
(2) Outcome of the home diet, establishing three subcategories: the unrestricted diet for milk and dairy products, OIT failure as complete avoidance of milk and dairy products, OIT still in progress with the intake of a defined and fixed milk quantity due to adverse symptoms and reactions at higher doses or refusal by the child.

### 2.2 Statistical analysis

Descriptive statistics are presented as frequencies and percentages for categorical variables and as mean, SD, median, minimum, and maximum values for numerical variables.

Pearson *χ* ^2^ test and Fisher exact test were calculated to evaluate associations between variables. Wilcoxon–Mann–Whitney *U* and Kruskal–Wallis tests were used to compare groups, while Spearman coefficients were employed to assess the correlation between continuous variables following the Shapiro test for normality check. The Youden index *J* was used to determine the optimal cutoff age by estimating a univariate logistic model. Logistic regression was estimated to estimate the association between the highest dose of milk ingested during the in‐hospital rush phase and several risk factors, whereas multinomial logistic regression was adopted to evaluate the association with long‐term outcome of OIT at home (three‐categories outcomes: the unrestricted diet for milk and dairy products; avoidance of milk and dairy products; oral immunotherapy). A significance level of 10% was applied as criteria of inclusion for risk factors in multiple regression analyses, based on previously estimated univariate models (results not shown). Conclusions were presented in terms of odds ratios (ORs) and relative risks (RRs) with a 95% confidence interval (95% CI). Analyses were run on Stata 16 and R software.^11^

## 3 RESULTS

From the 391 patients’ charts reviewed, 131 met the inclusion criteria for the retrospective study (Supporting Information: Figure 1S), with an average follow‐up of 64.08 months (12–143 months).

The clinical characteristics of the population included in the study are reported in Table 1 and Supporting Information: Table S1. Supporting Information: Tables 3S and 4S show the clinical characteristic included in the “in‐hospital rush phase” and in the “long term home‐phase OIT,” respectively.

**Table 1:** Clinical characteristics of the 131 included patients at admission

	Value (overall *N* = 131)
Sex	
Female	54 (41.2%)
Age of diagnosis of CMA	Months
Mean (SD)	5.89 (10.70)
Median [Min, Max]	5.00 [0, 12]
Age at the time of the “in‐hospital admission”	Years
Mean (SD)	8.13 (3.94)
Median [Min, Max]	6.00 [4.00, 22.0]
Associated food allergies	Value (%)
None	70 (51.9%)
One	46 (34.1%)
Two or more	19 (14.1%)
Comorbidities	Value (%)
None	27 (20.6%)
Wheezing	99 (75.57%)
Allergic rhinitis	3 (2.3%)
Atopic dermatitis	4 (3.0%)
Final CM dose during the hospitalization	ml
Mean (SD)	12.90 (7.41)
Median [Min, Max]	12.00 [0, 45]
Max CM dose during OIT at home	ml
Mean (SD)	107.02 (90.41)
Median [Min, Max]	82.5 [0, 250]

Abbreviations: CMA, cow milk allergy; NA, not available; s, specific.

### 3.1 The outcome of the in‐hospital “rush” phase

During the in‐hospital “rush” phase of the OIT, a part of the patients (25, 19.1%) presented mild reactions, while the majority presented one or more systemic reactions (in total 263 reactions, average 2 reactions per patient; Supporting Information: Figure S2).

In seven patients (5.3%), OIT had to be stopped during the in‐hospital “rush phase” because the first milk doses repeatedly induced allergic reactions, and 38 (29%) started home‐phase OIT with a dose of cow's milk less than 10 ml.

One patient in this series died of a fatal anaphylaxis after OIT failure, while being on CM avoidance in the last 2 years, notwithstanding immediate use of adrenaline self‐injector.

Two patients were diagnosed with eosinophilic esophagitis.

### 3.2 The outcome of the long‐term home‐phase OIT

All patients involved in the study were contacted regularly by a clinical investigator (by phone or email) to collect data on the OIT's home performance: of the 124 patients included in the study, 20 were not available for follow‐up (15.6%); therefore, follow‐up data are available for 104 patients.

Of these 104 patients, 45 (43.3%) were on an unrestricted diet (reached on average 22 months after the first admission to start OIT), while the rest continued to take a fixed dose of milk (29 patients, 22.1%) or had discontinued OIT (30 patients, 22.9%) due to reactions and eventual refusal to assume further milk.

The medium home dosage was 107.02 ml (range 0–250 ml), 19 patients (18.3%) reached the maximum dose of 250 ml of milk, and 8 children (7.7%) a dose below 10 ml.

Among the 104 patients in home OIT, three children (2.8%) showed no reaction; the remaining presented at least one reaction during the long‐term phase. The symptoms experienced at home were wheezing (58 patients, 55.8%), drowsiness (6, 5.8%), generalized urticaria (9, 8.6%), collapse (15, 14.4%), abdominal pain/vomit (3, 2.8%), dysphonia/dry cough (3, 2.8%), angioedema (3, 2%), and oral itching/perioral urticaria (1, 0.9%). Finally, 13 patients (12.5%) required an ED evaluation for allergic reactions during the home phase, and one of them was admitted to an intensive care unit.

The data obtained from this chart review were then analyzed regarding the two outcomes previously described (Table 2 and Supporting Information: Table 5S).

**Table 2:** Comparison of the demographic data with the outcome of the home diet

	Outcome of the home diet			
	Unrestricted diet (*N* = 45)	OIT in progress (build up or manteinance phase) (*N* = 29)	CM avoiding diet (*N* = 30)	*p *value
Sex				0.380
Female	16 (35.6%)	15 (51.7%)	12 (40.0%)	
Age of diagnosis of CMA	Months	Months	Months	0. 482
Mean (SD)	4.74 (3.68)	4.91 (2.67)	8.95 (21.18)	
Median [Min, Max]	4.00 [1.00, 24.0]	5.00 [1.00, 12.0]	6.00 [0, 12]	
Number of reactions				0.637
≤1	7 (15.6%)	1 (3.5%)	3 (10.7%)	
2–5	13 (28.9%)	9 (31.0%)	9 (32.1%)	
>5	24 (53.3%)	17 (58.6%)	16 (57.2%)	
NA	1 (2.2%)	2 (6.9%)	2 (6.7%)	
IM adrenaline during the home OIT				0.052
No	40 (88.9%)	19 (65.5%)	23 (76.7%)	
Yes	5 (11.1%)	10 (34.5%)	7 (23.3%)	
Associated food allergies				0.612
None	22 (48.9%)	16 (55.2%)	14 (46.7%)	
One	14 (31.1%)	11 (37.9%)	12 (40.0%)	
Two or more	9 (20.0%)	2 (6.9%)	4 (13.3%)	
Age at the time of admission	Years	Years	Years	0.011
Mean (SD)	7.07 (3.06)	8.07 (3.59)	10.13 (4.92)	
Median [Min, Max]	6.00 [4.00, 15.0]	7.00 [4.00, 16.0]	8.00 [5.00, 22.0]	
Oral itching/perioral urticaria				0.989
No	33 (73.3%)	22 (75.9%)	23 (76.7%)	
Yes	12 (26.7%)	7 (24.1%)	7 (23.3%)	
Rhinitis				0.060
No	38 (84.4%)	27 (93.1%)	19 (63.3%)	
Yes	7 (15.6%)	2 (6.9%)	11 (36.7%)	
Abdominal pain/vomit				0.414
No	24 (53.3%)	16 (55.2%)	16 (53.3%)	
Yes	21 (46.7%)	13 (44.8%)	14 (46.7%)	
Generalized urticaria				0.637
No	32 (71.1%)	19 (65.5%)	17 (56.7%)	
Yes	13 (28.9%)	10 (34.5%)	13 (43.3%)	
Wheezing				0.020
No	29 (64.4%)	14 (48.3%)	11 (36.7%)	
Yes	16 (35.6%)	15 (51.7%)	19 (63.3%)	
Drowsiness				1.000
No	44 (97.8%)	28 (96.6%)	29 (96.7%)	
Yes	1 (2.2%)	1 (3.4%)	1 (3.3%)	
Collapse				1.000
No	44 (97.8%)	29 (100%)	29 (96.7%)	
Yes	1 (2.2%)	0 (0%)	1 (3.3%)	
IM adrenaline during in‐hospital rush				0.210
No	45 (100%)	28 (96.6%)	28 (93.3%)	
Yes	0 (0%)	1 (3.4%)	2 (6.7%)	
Total reactions during the hospitalization				
Mean (SD)	1.59 (1.24)	1.89 (1.50)	2.94 (2.24)	0.032
Median [Min, Max]	1.00 [0, 5.00]	2.00 [0, 6.00]	3.00 [0, 7.00]	
NA	1 (2.2%)	1 (3.5%)	3 (10.0%)	
Final CM dose during the hospitalization	ml	ml	ml	0.024
Mean (SD)	15.09 (8.04)	11.96 (6.40)	10.02 (6.77)	
Median [Min, Max]	15 [3, 45]	10 [0.4, 20]	11 [0, 25]	
Max CM dose during the home OIT	ml	ml	ml	<0.001
Mean (SD)	177.04 (80.05)	87.81 (62.46)	34.33 (53.69)	
Median [Min, Max]	200 [10, 250]	70 [4.4, 90]	6 [0, 8]	
Need for hospital admission				0.354
No	41 (91.1%)	26 (89.7%)	23 (76.7%)	
Yes	4 (8.9%)	3 (10.3%)	6 (20.0%)	
NA	0 (0%)	0 (0%)	1 (3.3%)	

Abbreviation: CM, cow milk; CMA, cow milk allergy; NA, not available; OIT, oral immunotherapy.

Older age was correlated with the probability of having a final amount of milk ≥10 ml after the in‐hospital rush phase. The optimal cut‐off identified with the Youden Index was 6 years (Supporting Information: Figure 3S).

Concerning the long‐term diet outcome, patients were divided into three distinct categories: unrestricted diet for milk and dairy products, CM avoiding diet, and patients in the build‐up or maintenance phase of OIT. A significant association (*p* < .05) was found with OIT failure and age, as with increasing age the likelihood of having a successful OIT home phase decreased. In this case, the optimal cut‐off identified with the Youden Index was 10 years (Supporting Information: Figures 4S and 5S). Furthermore, other significant risk factors for OIT failure were IM adrenaline usage, sIgE to β‐lactoglobulin, the onset of wheezing during home OIT, and the total number of reactions experienced (Table 3 and Supporting Information: Table 5S).

**Table 3:** Multivariate analysis of the RR of not having an unrestricted diet for milk and dairy products

Outcome of the home diet (calculated regards the unrestricted diet for milk and dairy products)	Still in progress	CM avoiding diet
Population characteristics	RR (CI 95%)	RR (CI 95%)
Age at the time of admission (>10 years)	0.61 (0.15; 2.49)	3.29 (0.85; 12.73)*
Milk sIgE (285 vs. 458)	1.00 (0.99; 1.01)	1.00 (0.99; 1.01)
ß‐Lactoglobulin sIgE (72.9 vs. 208)	1.00 (0.99; 1.01)	1.01 (1.00; 1.02)*
Casein sIgE (292 vs. 489)	1.00 (0.99; 1.01)	1.00 (0.99; 1.01)
Rhinitis (no/yes)	0.46 (0.08; 2.76)	2.12 (0.56; 8.03)
Wheezing (yes/no)	3.78 (1.01; 14.12)**	1.93 (0.49; 7.61)
Total reactions during hospitalization (1.55 vs. 3.32)	0.95 (0.63; 1.43)	1.54 (1.02; 2.32)**
Use of IM epinephrine (yes/no)	5.47 (1.39; 21.45)**	1.60 (0.33; 7.73)

Abbreviations: CI, confidence interval; CM, cow milk; RR, relative ratio.

*p < .10, **p < .05.

Multilinear regression confirmed the association between the OIT discontinuation and the number of reactions, sIgE to β‐lactoglobulin, and a respiratory reaction with wheezing (Supporting Information Table 6S).

The use of IM epinephrine was also associated with the risk of having the OIT still in progress.

Finally, the association between final milk dosage at the time of discharge, as an independent variable, and an unrestricted diet was assessed as a dependent variable, although statistical significance was not achieved. The results suggested a possible association between the two outcomes considered (RR, [95% CI]: 2.33, [0.85; 6.42]. A dose of milk at the time of discharge of less than 10 ml increases the risk of discontinuing OIT by 2.33 times (Supporting Information: Figure 6S).

## 4 DISCUSSION

In this study wheezing reactions during the in‐hospital phase admission, discharge with a dose below 10 ml, use of IM epinephrine, and older age (over 10 years) were all risk factors for OIT discontinuation.

In this series, 43.3% of 104 patients with severe CMA undergoing OIT were able to introduce unrestricted raw milk, while 22% of them tolerated a variable amount of milk. This latter percentage is still relevant since the maintenance of milk intake could decrease the risk of extremely severe reactions after accidental contamination. Furthermore, a partial tolerance has been related to a better quality of life and could hasten a future full tolerance.^12^

These findings are similar to the existing literature compared to cohorts of patients with similar SPT and sIgE range values.^9^, ^13^, ^14^, ^15^, ^16^

In this study, a relevant proportion of patients displayed different degrees of bronchial reactivity (74, 56.5%), also related to a higher proportion of OIT failure during the maintenance phase.^9^

Even considering the severity of our study group, OIT efficacy remains fair.

On the other hand, this study confirmed the high prevalence of several kinds of reactions, to the point that 31.3% of patients dropped, primarily due to the presence of recurrent and severe symptoms.

For this specific reason, we believe it may be relevant to focus the attention on the risk factors that can predict OIT failure. Moreover, children and adolescents at the highest risk of failure should be considered eligible for different approaches, such as the use of omalizumab.^17^

In this study an older age was significantly related to a higher risk of OIT failure over time. This finding can be explained by a more difficult possibility for adolescent patients to reconcile OIT assumption at home with their social commitments, such as sports and leisure activities. Home intake should take place far from physical exercise due to the risk of exercise‐induced allergic reactions and allowing a 2‐h home observation. The adolescents may be poorly compliant to these daily limitations, exacerbated by the fear of adverse reactions. Evidence from the literature shows that the life quality of some of these patients may be significantly worsened by the development of anxiety related to allergen forced intake.^18^ Overall, an assumption‐related anxiety can be even worse than an antigen avoiding one. Finally, adolescents with failed OIT may be at higher risk of very severe reactions when compared to peers with ongoing milk intake.^19^

In this perspective the development of protocols showing the higher effectiveness and safety of OIT at an early age appears encouraging and deserves further investigations.^10^, ^20^

This study has limitations: patients did not undergo a previous food challenge due to their severe and convincing history and high IgE levels, but almost all of them displayed reactions to milk. Moreover, it is a retrospective study without a control group to better investigate the natural resolution of milk allergy. However, the patients' age and severity suggest the unlikelihood of a natural resolution of milk allergy over time. Finally, 16.1% of patients were lost during follow‐up.

The point of strength is that this is one of the largest cohorts evaluating the impact of the safety and efficacy of long‐term milk OIT, along with another study,^9^, ^21^ and the only one which investigates this outcome in a selected population with high sIgE levels.

## 5 CONCLUSION

In conclusion, this study shows that being discharged after the in‐hospital phase with less than 10 ml of pure milk after a rush in the hospital phase, presenting reactions with wheezing, needing IM epinephrine for reactions, and being older than 10 years are predictive factors of poor OIT outcome.

The provided phenotype of patients with a severe milk allergy who are more likely to fail OIT can help pediatricians to provide to their parents the elements to properly decide the most appropriate approach.

## AUTHOR CONTRIBUTIONS

Elisa Benelli, Andrea Trombetta, Laura Badina, Stefanny Andrade, and Antonio Prisco were involved in concept and design, in drafting the article and revising it critically for important intellectual content; and they finally approved the version to be published. Eugenio Traini and Giulia Zamagni were involved in statistical analyses, and they finally approved the version to be published. Egidio Barbi and Irene Berti were involved in drafting the article and revising it critically for important intellectual content, and they finally approved the version to be published.

## CONFLICT OF INTEREST

The authors declare no conflict of interest.

## ETHICS STATEMENT

The study was approved by the Institutional Review Board, IRB 05/17.

## Supporting information

Supporting Information.

Supporting Information.

Supporting Information.

Supporting Information.

Supporting Information.

Supporting Information.

Supporting information.

## Data Availability

All data generated or analyzed during this study are included in this published article.
