# Supplementary figures and images for: Risk factors for discontinuing oral immunotherapy in children with persistent cow milk allergy
Source: Immun Inflamm Dis. 2022 Jun 20;10(7):e668. doi: 10.1002/iid3.668 (PMC9208286; doi:10.1002/iid3.668)

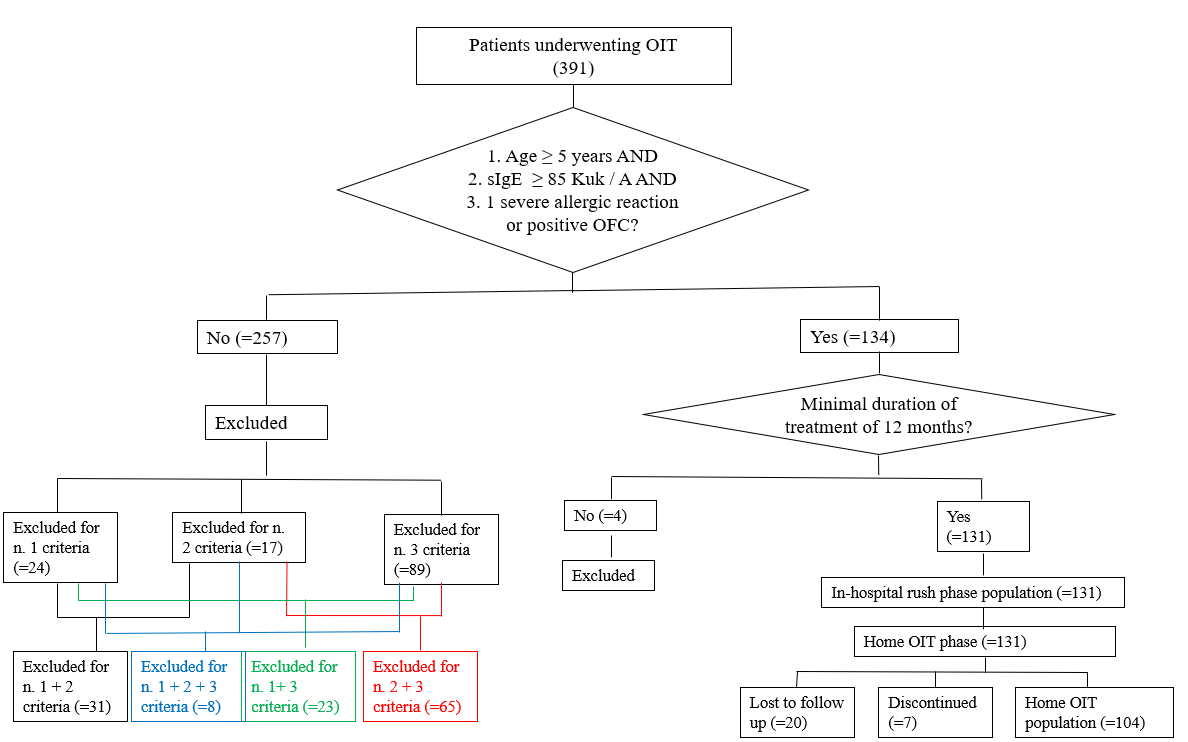

Supplement: Supplementary file 1 — Supporting Information. [file IID3-10-e668-s005.PNG]

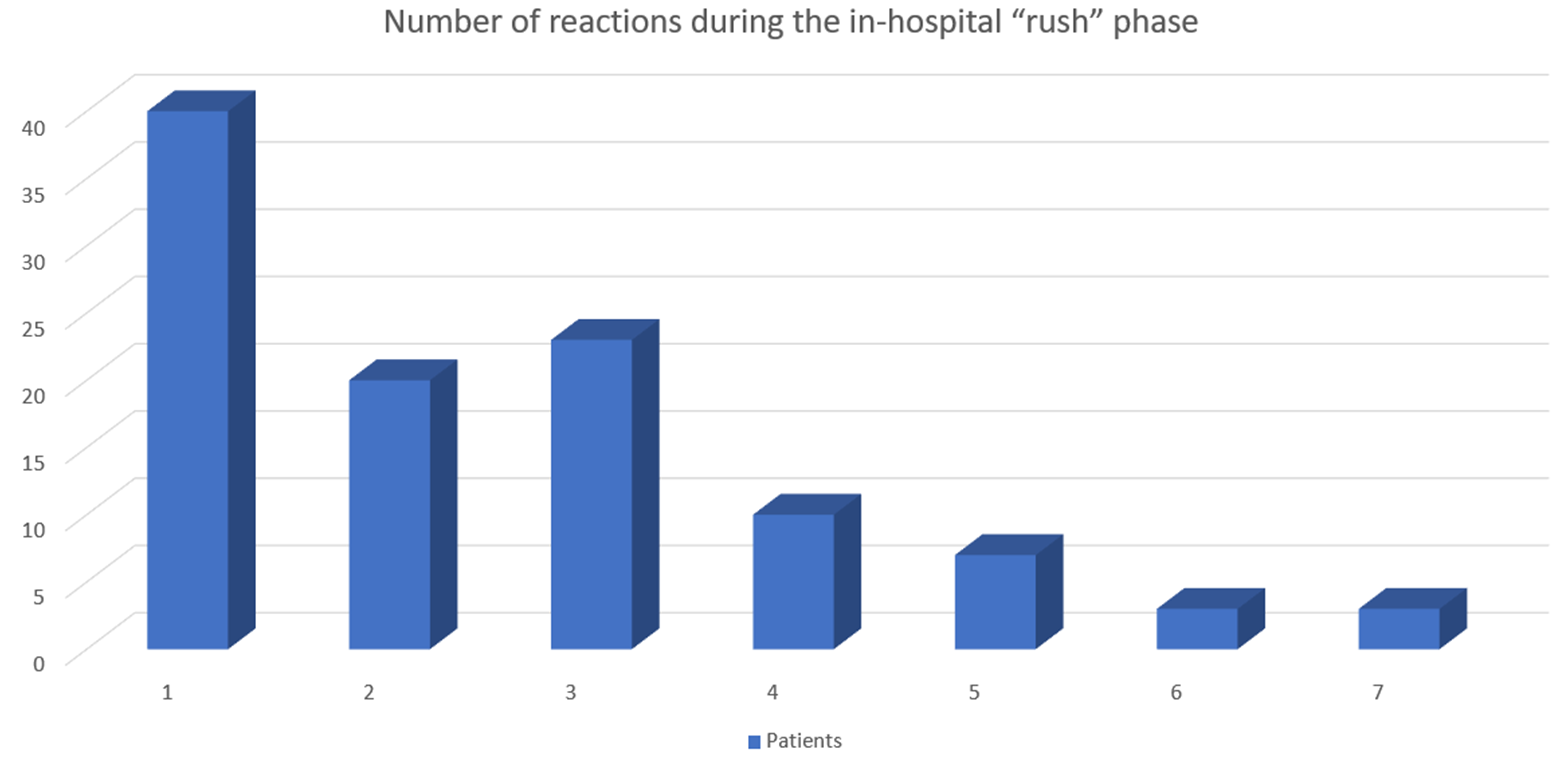

Supplement: Supplementary file 2 — Supporting Information. [file IID3-10-e668-s002.PNG]

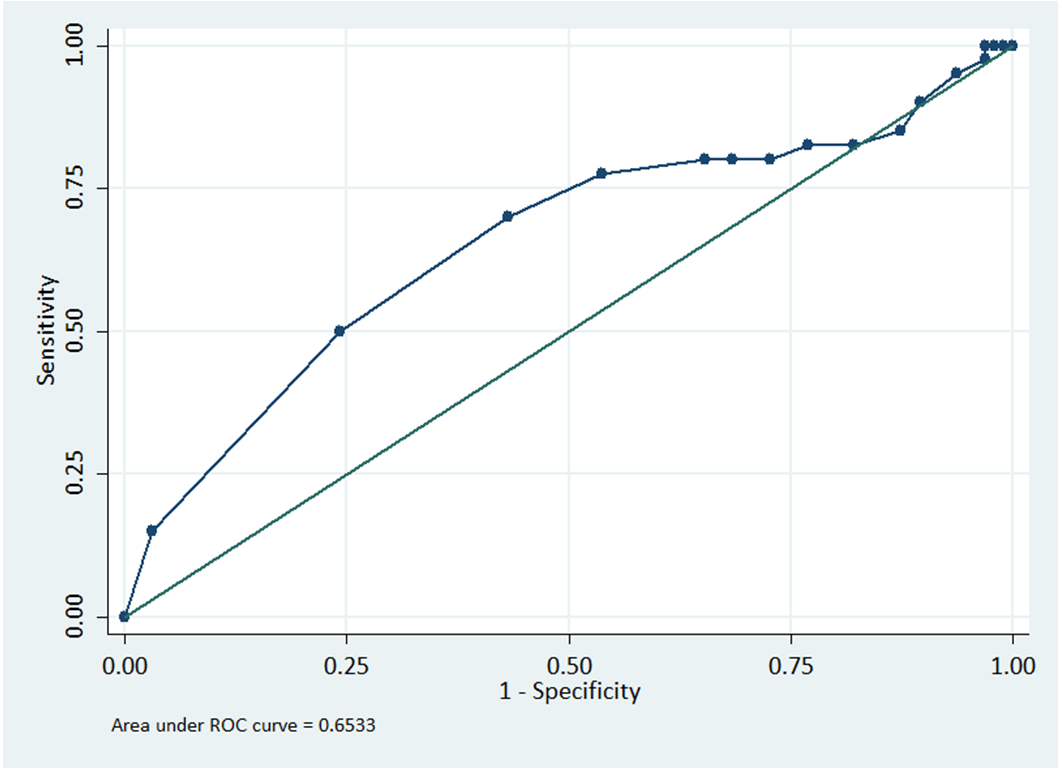

Supplement: Supplementary file 3 — Supporting Information. [file IID3-10-e668-s007.PNG]

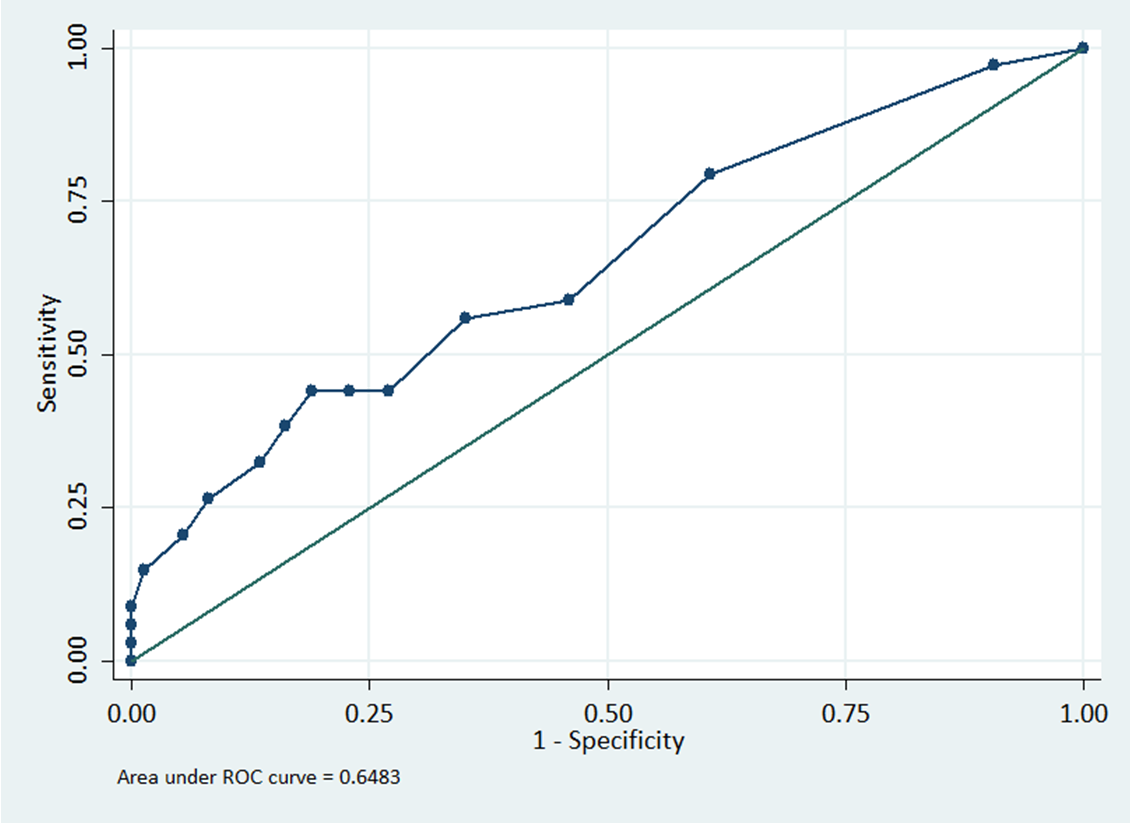

Supplement: Supplementary file 4 — Supporting Information. [file IID3-10-e668-s006.PNG]

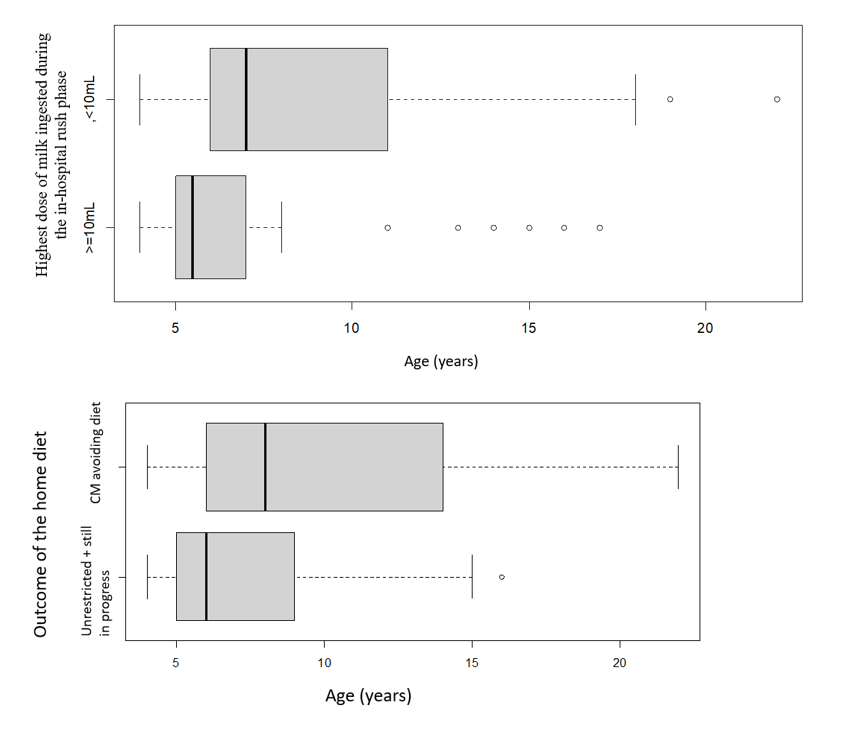

Supplement: Supplementary file 5 — Supporting Information. [file IID3-10-e668-s003.PNG]

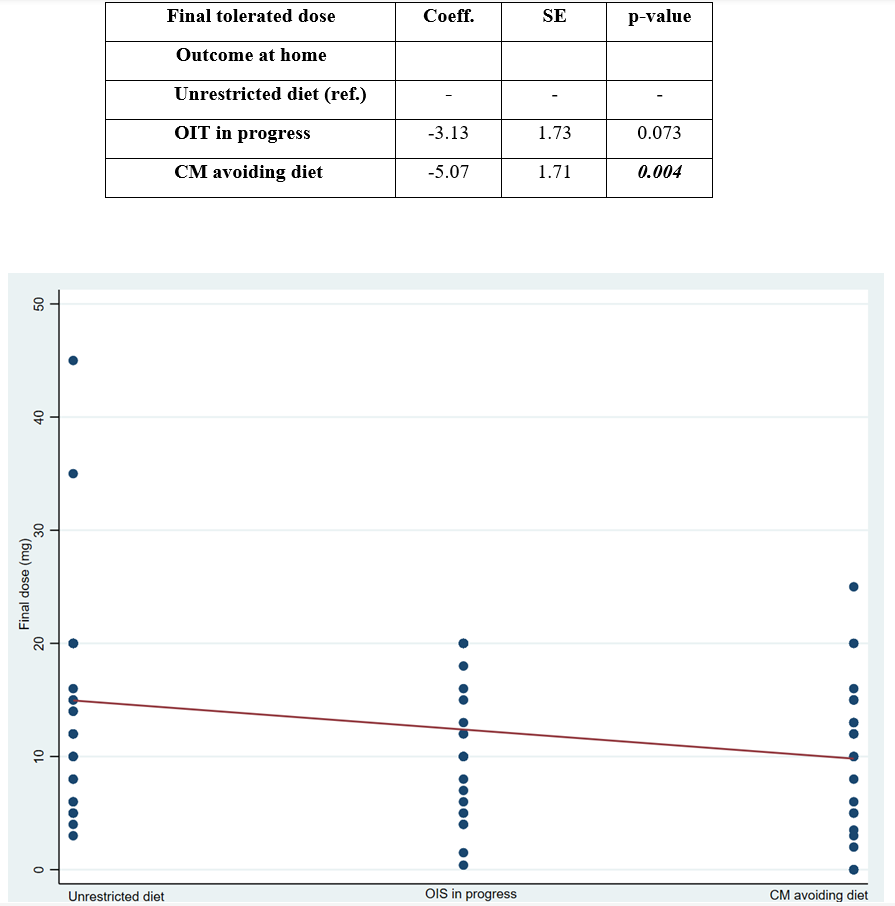

Supplement: Supplementary file 6 — Supporting Information. [file IID3-10-e668-s004.PNG]
