# Supplementary material for: Risk factors for discontinuing oral immunotherapy in children with persistent cow milk allergy
Source: Immun Inflamm Dis. 2022 Jun 20;10(7):e668. doi: 10.1002/iid3.668 (PMC9208286; doi:10.1002/iid3.668)
Supplement: Supplementary file 7 — Supporting information. [file IID3-10-e668-s001.docx]

**Table 1S: Specific Oral Tolerance Induction (SOTI) protocol**

Doses were administered every 1 hour on the first days and every 2 hours on the other days.

^*^1 mL = 20 drops

| **Day** | **Dilution (mL of cow’s milk)** | **Dose (mg of cow’s milk protein)** |
| --- | --- | --- |
| 1 | 1 drop^*^ of cow’s milk in 10 mL of water | 0.25 mg, 0.5 mg, 33 mg, 66 mg, 165 mg, 330 mg |
| 2 | 5 drops^*^ of cow’s milk in 20 mL of water | 66 mg, 132 mg, 264 mg, 528 mg |
| 3 | 1 mL of cow’s milk in 20 mL of water | 66 mg, 132 mg, 264 mg, 396 mg |
| 4 | 3 mL of cow’s milk in 20 mL of water | 99 mg, 198 mg, 297 mg, 330 mg |
| 5 | 10 mL of cow’s milk in 20 mL of water | 99 mg, 198 mg, 297 mg |
| 6 | 10 mL of cow’s milk in 10 mL of water | 99 mg, 198 mg, 297 mg |
| 7 | Pure cow’s milk | 66 mg, 132 mg, 198 mg |
| 8 | Pure cow’s milk | 132 mg, 231 mg, 330 mg |
| 9 | Pure cow’s milk | 264 mg, 396 mg, 495 mg |
| 10 | Pure cow’s milk | 429 mg, 528 mg, 660 mg |

**Table 2S: Increasing protocol after the in-hospital phase**

After the in-hospital phase, patients were instructed to follow a specific increasing protocol adapted to their outcome at the discharge.

| **Tolerance dose at discharge (mg of cow’s milk protein)** | **First increase at home (mg of cow’s milk protein)** | **Following increases at home (mg of cow’s milk protein)** | | | |
| --- | --- | --- | --- | --- | --- |
| > 495 mg | 66 mg every 2-3 days up to 1.98 g | 165 mg every 2-3 days up to 8.25 g | | | |
| < 495 mg but > 165 mg | 33 mg every 5-7 days up to 990 mg | 66 mg every 2-3 days up to 1.98 g | | 165 mg every 2-3 days up to 8.25 g | |
| < 165 mg | 16.5 mg every 7-10 days up to 165 mg | 33 mg every 5-7 days up to 990 mg | 66 mg every 2-3 days up to 1.98 g | | 165 mg every 2-3 days up to 8.25 g |

**Table 3S**. In-hospital “rush” phase population characteristics.

| **Oral itching/perioral urticaria** |  |
| --- | --- |
| No | 99 (75.6%) |
| Yes | 32 (24.4%) |
| **Rhinitis** |  |
| No | 109 (83.2%) |
| Yes | 22 (16.8%) |
| **Abdominal pain/vomit** |  |
| No | 68 (51.9%) |
| Yes | 63 (48.1%) |
| **Generalised urticaria** |  |
| No | 86 (65.7%) |
| Yes | 45 (34.3%) |
| **Wheezing** |  |
| No | 64 (48.8%) |
| Yes | 67 (51.2%) |
| **Drowsiness** |  |
| No | 128 (97.7%) |
| Yes | 2 (1.5%) |
| **Collapse** |  |
| No | 129 (98.5%) |
| Yes | 2 (1.5%) |

**Table 4S:** Long-term phase-home OIT population characteristics. NA: not available

| **Reactions during the home OIT** |  |
| --- | --- |
| None | 16 (12.2%) |
| Oral itching/perioral urticaria | 4 (3.0%) |
| Generalised urticaria | 9 (6.9%) |
| Wheezing | 74 (56.5%) |
| Drowsiness | 7 (5.3%) |
| Rhinitis | 6 (4.6%) |
| Abdominal pain/vomit | 6 (4.6%) |
| NA | 9 (6.9%) |
| **Need for hospital admission** |  |
| No | 109 (83.2%) |
| Yes | 14 (9.9%) |
| NA | 9 (6.9%) |

**Table 5S:** Comparison of the clinical demographic data with the highest dose of milk ingested during the in-hospital rush phase. CMA= cow milk allergy; IM= intramuscular; NA= not applicable; OIT= oral immunotherapy

|  | **Highest dose of milk ingested during the in-hospital rush phase** | | **p-value** |
| --- | --- | --- | --- |
|  | **(<10mL)**  **(N=38)** | **(≥10mL)**  **(N=93)** |  |
| **Sex** |  |  | 1.000 |
| Female | 16 (42.1%) | 38 (40.9%) |  |
| **Age of diagnosis of CMA** | **months** | **months** | 0.438 |
| Mean (SD) | 7.43 (18.90) | 5.26 (4.10) |  |
| Median [Min, Max] | 4.5 [1.00, 120] | 5.0 [0, 30.0] |  |
| **Number of reactions** |  |  | 0.844 |
| ≤ 1 | 3 (7.9%) | 11 (11.8%) |  |
| 2-5 | 12 (31.5%) | 26 (28.0%) |  |
| > 5 | 21 (55.3%) | 51 (54.8%) |  |
| NA | 2 (5.3%) | 5 (5.4%) |  |
| **Most severe reaction before in-hospital rush phase** |  |  | 0.055 |
| None | 1 (2.6%) | 2 (2.1%) | 1.000 |
| Oral allergy syndrome | 0 (0%) | 1 (1.2%) | 1.000 |
| Generalized orticaria | 1 (2.6%) | 9 (9.7%) | 0.279 |
| Wheezing | 24 (63.2%) | 49 (52.7%) | 0.327 |
| Abdominal pain | 2 (5.3%) | 4 (4.3%) | 0.565 |
| Collapse | 1 (2.6%) | 17 (18.3%) | 0.046 |
| Dysphonia/dry cough | 2 (5.3%) | 2 (2.1%) | 0.579 |
| Angioedema | 1 (2.6%) | 3 (3.2%) | 1.000 |
| Drowsiness | 5 (13.2%) | 3 (3.2%) | 0.022 |
| NA | 1 (2.6%) | 3 (3.2%) | - |
| **IM epinephrine** |  |  | 0.302 |
| No | 28 (73.7%) | 76 (81.7%) |  |
| Yes | 10 (26.3%) | 17 (18.3%) |  |
| **Associated food allergies** |  |  | 0.451 |
| None | 23 (60.5%) | 44 (47.3%) |  |
| One | 11 (29.0%) | 34 (36.6%) |  |
| Two or more | 4 (10.5%) | 15 (16.1%) |  |
| **Comorbidities** |  |  | 0.171 |
| None | 9 (23.7%) | 18 (19.3%) | 0.578 |
| Allergic rhinitis | 1 (2.6%) | 2 (2.2%) | 1.000 |
| Atopic dermatitis | 1 (2.6%) | 3 (3.2%) | 1.000 |
| Wheezing | 27 (71%) | 70 (75.3%) | 0.050 |
| **Age at the time of admission** | **years** | **years** | 0.010 |
| Mean (SD) | 7.26 (3.91) | 8.48 (3.91) |  |
| Median [Min, Max] | 5.50 [4.00, 17.0] | 7.00 [4.00, 22.0] |  |
| **Oral itching/perioral urticaria** |  |  | 0.374 |
| No | 31 (1.6%) | 68 (73.1%) |  |
| Yes | 7 (18.4%) | 25 (26.9%) |  |
| **Rhinitis** |  |  | 0.444 |
| No | 30 (79.0%) | 79 (85.0%) |  |
| Yes | 8 (21.0%) | 14 (15.0%) |  |
| **Abdominal pain/vomit** |  |  | 0.179 |
| No | 16 (42.1%) | 52 (55.9%) |  |
| Yes | 22 (57.9%) | 41 (44.1%) |  |
| **Generalised urticaria** |  |  | 0.110 |
| No | 21 (55.3%) | 65 (69.9%) |  |
| Yes | 17 (44.7%) | 28 (30.1%) |  |
| **Wheezing** |  |  | <0.001 |
| No | 9 (23.7%) | 55 (59.1%) |  |
| Yes | 29 (76.3%) | 38 (40.9%) |  |
| **Drowsiness** |  |  | 1.000 |
| No | 37 (97.4%) | 91 (97.9%) |  |
| Yes | 1 (2.6%) | 2 (2.1%) |  |
| **Collapse** |  |  | 0.083 |
| No | 33 (94.7%) | 93 (100%) |  |
| Yes | 2 (5.3%) | 0 (0%) |  |
| **IM epinephrine during the in-**  **hospital rush** |  |  | 0.202 |
| No | 36 (94.7%) | 92 (98.9%) |  |
| Yes | 2 (5.3%) | 1 (1.1%) |  |
| **Total reactions during hospitalization** |  |  | <0.001 |
| Mean (SD) | 3.32 (1.85) | 1.55 (1.40) |  |
| Median [Min, Max] | 3.00 [0, 7.00] | 1.00 [0, 7.00] |  |
| NA | 4 (10.5%) | 2 (2.1%) |  |
| **Final CM dose during the hospitalization** | **mL** | **mL** | <0.001 |
| Mean (SD) | 4.38 (2.68) | 16.38 (5.7) |  |
| Median [Min, Max] | 5 [0, 8] | 16 [10, 45] |  |
| **Max CM dose during OIT at home** | **mL** | **mL** | <0.001 |
| Mean (SD) | 63.37 (79.55) | 124.88 (88.87) |  |
| Median [Min, Max] | 24 [0, 250] | 100 [5, 250] |  |
| NA | 2 (5.3%) | 5 (5.4%) |  |
| **Need for hospital admission** |  |  | 0.684 |
| No | 31 (81.6%) | 78 (83.9%) |  |
| Yes | 3 (7.9%) | 10 (10.8%) |  |
| NA | 4 (10.5%) | 5 (5.4%) |  |

**Table 6S:** Multivariate analysis of the OR of having a dosage of milk lower than 10mL during the “in-hospital rush phase”.**p<0.05*

| **Dosage of milk ingested during the in-hospital rush phase < than 10mL** | **OR** | **CI 95%** |
| --- | --- | --- |
| Age at the time of admission (6 years) | 0.32* | 0.12; 0.88* |
| Alfa- lactalbumin sIgE (266 vs 169) | 1.00 | 0.99; 1.01 |
| Cow’s milk sIgE (404 vs 239) | 1.00 | 1.00; 1.01 |
| Wheezing | 2.34 | 0.82; 6.71 |
| Generalized urticaria | 1.04 | 0.37; 2.92 |
| Number of reactions during the in-hospital phase (3.32 vs 1.55) | 1.64* | 1.18; 2.29* |
